# To eat or to care? Factors shaping parental or infanticidal behaviours in male poison frogs during territory takeover

**DOI:** 10.1186/s12983-025-00567-1

**Published:** 2025-07-02

**Authors:** Leïla Perroulaz, Lauriane Bégué, Eva Ringler

**Affiliations:** 1 Institute of Biology, University of Neuchâtel, 2000 Neuchâtel, Switzerland; 2 Division of Behavioural Ecology, Institute of Ecology and Evolution, University of Bern, 3032 Hinterkappelen, Switzerland

**Keywords:** Cannibalism, Parental care, Tadpole transport, Reproductive behaviour, Clutch recognition, Mating activity

## Abstract

**Background:**

Parental care is costly for the caregiver. Therefore, parents should be able to discriminate between their own and conspecific offspring to avoid costly misdirected care. Infanticide, the intentional killing of conspecific young by adult individuals, occurs in many animal taxa. It has been shown to have several benefits for the perpetrator, such as increasing mating opportunities, ensuring dominance, or reducing a competitor’s fitness; but infanticide may also minimise risks of misdirected parental care. Previous studies in *Allobates femoralis*, a poison frog with parental care, have shown that males transport all tadpoles present in their territory to water bodies, regardless of whether they have sired the clutch or not. However, when taking over a new territory, males cannibalise clutches from the previous territory holder. These findings raise the question as to which factors actually shape parental care and infanticidal behaviour in male *A. femoralis* after territory takeover. To answer this question, we designed a laboratory experiment, in which we tested males with different territorial status and recent mating activity. We recorded tadpole transport and cannibalism and compared the occurrence of these two behaviours across our different experimental conditions.

**Results:**

We found that territory ownership, relatedness to clutches, and possibly also recent mating activity influenced parental behaviours. However, we were unable to clearly disentangle the factors influencing cannibalistic behaviours. Our results also confirmed that males use territorial recognition to discriminate between their own and unrelated offspring, and that they commit infanticide likely to avoid misdirected parental care.

**Conclusions:**

Transport and cannibalism appear to be impacted by several factors in different ways. We found that the territorial status and relatedness to the clutch both influence parental behaviours in male poison frogs, whereas the factors influencing infanticidal behaviours remain unclear. Therefore, transport and cannibalism appear to be two independent processes, and factors influencing one behaviour do not necessarily affect the other. Further studies should investigate associated neuroendocrine changes, to better understand the mechanisms underlying parental and infanticidal behaviour in poison frogs. Our findings suggest that the decision-making processes involved in tadpole transport and clutch cannibalism appear to be more complex than previously thought.

**Supplementary Information:**

The online version contains supplementary material available at 10.1186/s12983-025-00567-1.

## Background

Parental care encompasses all aspects that enable parents to increase the fitness of their offspring, such as nest construction, preparing and maintaining a proper rearing environment, predator defence, teaching, feeding, and transport [1]. It is a costly investment by the parent, often compromising future reproductive opportunities [1]. However, parental care is beneficial to the recipients of that care, as they have a better chance of survival and faster growth while being cared for. Higher offspring survival will then indirectly benefit the closely related caregivers, by increasing their inclusive fitness [2].

As parental care is costly, in most cases it is not adaptive for parents to care for offspring that are not their own. Therefore, in many animal taxa, mechanisms have evolved to be able to discriminate between one’s own offspring and the offspring of others: animals may use direct cues, such as chemical [3, 4], acoustic [5, 6] or visual information [7, 8]; or also indirect cues, such as spatial location [9] or time since larval hatching [10].

There are two types of errors that can occur with respect to offspring recognition [11]. On the one hand, parents may mistake an unrelated offspring for their own. Given the costly nature of parental care, it is not optimal for a parent to care for an unrelated offspring, as they would not benefit from an increase in their inclusive fitness and the eventual costs to their own growth and viability could negatively affect future reproductive success [2]. On the other hand, parents may also fail to correctly recognise their own offspring and therefore fail to provide the appropriate level of care to them [11]. This failure to recognise offspring can be costly for the parents, as it results in a decrease in their inclusive fitness [2]. These two potential sources of error have led to the development of parental strategies based on decision rules to minimise misidentification [3]. Misidentification is especially costly in contexts where species exhibit high levels of aggression towards unrelated young, potentially resulting in infanticide.

Infanticidal behaviour, both towards unrelated as well as own offspring is common in a wide range of species, including mammals [12–14], birds [15–17], fish [18–20], reptiles [21, 22], amphibians [23, 24] and arthropods [25–27]. There are several types of benefits a perpetrator may gain from exhibiting infanticidal behaviour [28]. Firstly, by eating the offspring, the individual gains nutritional benefits (predation hypothesis [28]). Indeed, cannibalism has been reported to increase when food is scarce [29, 30]. Moreover, an infanticidal conspecific eliminates competitors for resources that they may use for production or rearing of their own offspring, like access to natal territories or food (resource competition hypothesis [28]). Parents may also kill offspring of other conspecifics so that their own offspring have access to more food or other resources [31]. In the case of food limitation, some parents with a large number of offspring may give the younger sibling to feed the older ones [32] (parental manipulation hypothesis [28]). In addition, infanticide of non-direct descendants reduces reproductive output from sexual competitors and may increase parents’ reproductive opportunities (sexual selection hypothesis [28]). In some species, the loss of an infant may shorten the inter-ovulation period in females, thus allowing males to reproduce earlier [33]. Finally, there is some evidence that infanticide may help to prevent misdirected parental care [34, 35].

In amphibians, parental care is highly diverse and occurs across all three orders with over 30 different forms of parental care currently described [36]. The transition from aquatic to terrestrial habitats for reproduction led to a diversification of the reproductive modes, and parental care presumably facilitated this transition. In contrast to the high diversity of care, infanticide mainly occurs in the form of clutch cannibalism. In some salamanders, caring males selectively eat infected eggs to prevent infection from spreading to the rest of the clutch [37, 38]. There are only a few records of infanticide in frogs [23, 24, 39–42]. So far, the contexts in which this cannibalism occurs are quite diverse, and therefore we have limited understanding of the factors that drive clutch cannibalism in amphibians.

*Allobates femoralis* is a small diurnal Neotropical poison frog that has a pan-Amazonian distribution with isolated local populations [43, 44]. Males are highly territorial, announcing and defending their territory against intruders [45]. Females show site fidelity, but do not display any aggression against either male or female conspecifics [46]. Both males and females are polygamous and mate with multiple partners during the prolonged breeding season [47]. Mating takes place within the male’s territory, where the female deposits eggs in the leaf litter and the male fertilises them externally [46, 48]. When the eggs hatch, about three weeks after laying, the males transport the tadpoles on their backs to water bodies, usually located outside their territory, where the tadpoles complete their development until metamorphosis [49]. Field observations have revealed that males sometimes cannibalise foreign clutches, and an experiment conducted under laboratory conditions demonstrated that such cannibalism primarily occurs after taking over a new territory [40]. In turn, previous research in *A. femoralis* has also shown that once males have established territories, they transport all clutches located inside their territory to provided water bodies, regardless of their exact location and whether they have actually sired them or not [50]. What remains unknown is when and how this switch from being cannibalistic to being indiscriminately parental occurs.

The present study thus aimed to investigate the factors shaping parental care and cannibalistic behaviours in male *A. femoralis*. If territory novelty is the main factor shaping infanticidal behaviour, we expect that with increasing time spent in a territory, cannibalism rates would decrease, and parental care would increase. In addition, if recent mating activity is the main factor for eliciting parental behaviours, we would expect that shortly after mating cannibalism rates would decrease and parental care would increase, regardless of time spent in a novel territory.

## Methods

### Housing conditions

All frogs used in the present study are part of the laboratory population housed in the animal facilities at the Hasli Ethological station of the University of Bern (housing permits: BE 4/11 and BE 4/2022). The frogs are housed in breeding pairs in two different rooms, in terrariums of 60 × 40 × 40 cm and 50 × 40 × 40 cm size. Temperature, humidity and light are automatically controlled to be similar to natural conditions in French Guyana. The bottom of the terrarium is covered with expanded clay pebbles, over which autoclaved oak leaves are disposed. Each terrarium contains plants, half a coconut shell for shelter, a log for perching, and a water bowl. The sides and back of the terrarium are partially covered with xaxim (tree fern stems) and cork mats. A curtain covers the front side, to prevent visual contact across terrariums and minimise external disturbance. The frogs are fed wingless fruit flies dusted with vitamin powder twice a week.

### Experimental design

**Fig. 1:**
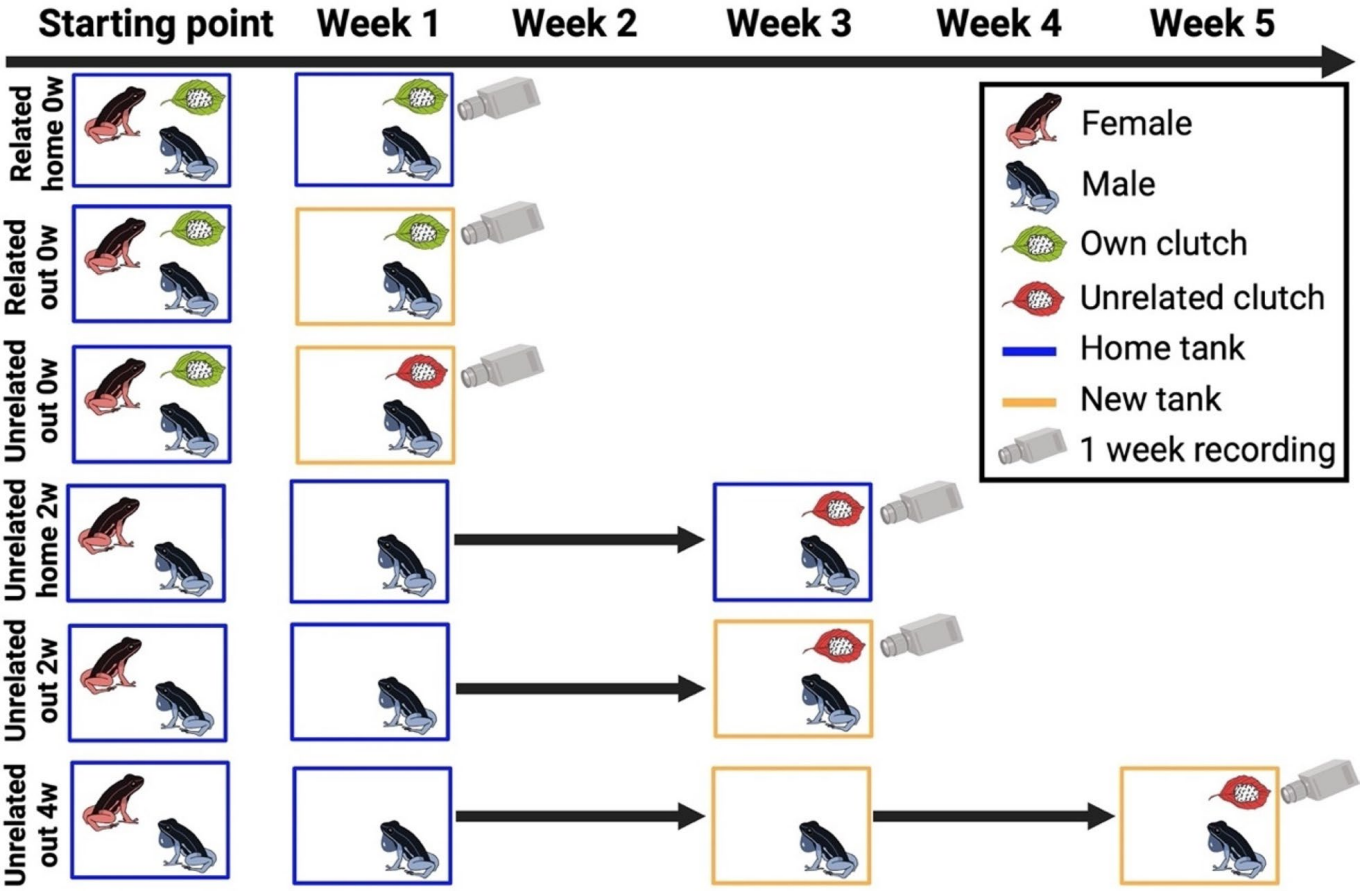
Experimental design. The starting point corresponds to the normal housing conditions, where frogs are housed in pairs. Some of these pairs have developing clutches inside their terrarium (indicated in green as “own clutch”), while some others have not. Individual males were semi-randomly assigned to one of the six conditions. See the main text for a detailed description of the conditions. (Scheme created by Leïla Perroulaz on BioRender)

We conducted experiments between September 2023 and April 2024. We tested a total of 90 males across six different conditions (Fig. 1), and only included males that had already successfully sired a clutch at least once. Each male was tested only once and assigned to one of the following six conditions. In Related home 0w, the female is removed from the terrarium and the male is recorded right after (zero week) in his home terrarium (home), with his own clutch (related) present. If the male had multiple clutches at the time of the experiment, the non-focal ones were removed from the terrarium. In Related out 0w, the male as well as his own clutch (related) are moved and recorded right after (zero week) in a new terrarium (out). In Unrelated out 0w, the male is moved and recorded right after (zero week) in a new terrarium (out) where an unrelated clutch, i.e. a clutch from another male, is placed. In Unrelated home 2w, the female is removed from the terrarium, and the male is kept isolated, i.e. without any mating partner, for two weeks. Then, an unrelated clutch is placed together with the male in his home terrarium. In Unrelated out 2w, the female is removed, and the male is isolated as in the previous condition. After two weeks, the male is placed in a new terrarium (out) where an unrelated clutch is present. In Unrelated out 4w, the male is isolated as described above. After two weeks, the male is transferred to a new terrarium (out). After two more weeks (i.e., four weeks in total), an unrelated clutch is added to the new terrarium. Males with an own clutch present at the time of the experiment were randomly assigned to one of the first three conditions (Related home 0w, Related out 0w or Unrelated out 0w), balancing the number of males to have fifteen individuals per condition. Males without an own clutch at the start of a given trial were assigned to one of the three last conditions (Unrelated home 2w, Unrelated out 2w or Unrelated out 4w). We did not test for all possible combinations of territorial status, reproductive state, timing, and relatedness of focal clutches, as some options were biologically not possible (Related out 4w). Moreover, to keep sample sizes as minimal as possible, we did not include some conditions that were already tested in previous studies (see [40, 50]).

All clutches used in the experiment were at similar developmental stage, about two weeks after oviposition (stage R-S [51]). The number of tadpoles per clutch did not significantly differ between the experimental conditions (Kruskal–Wallis rank sum test, Chisq = 4.59, df = 5, *p* = 0.47).

Frogs were caught in plastic zip-lock bags when transported between terrariums. All males were caught and kept in bags for a few minutes prior to recording, to prevent variations in handling effects across trials. Clutches were moved together with the leaves on which they were deposited, in all conditions. Leaves with clutches were placed at the front of the experimental terrarium, opposite the water bowl.

Females that had to be removed from their own terrarium were placed in empty terrariums for the duration of the trial. At the end of each trial, all frogs were returned to their home terrariums.

Trials were filmed from 8 am to 8 pm for one week, using surveillance cameras (Panasonic WV-U1132 i-PRO 2MP Varifocal Lens Indoor Box Network Camera) placed on top of the terrariums, making sure that the clutch was clearly visible. These cameras were connected to the Genetec video surveillance software [52].

### Video coding

Video analysis was done using the Boris software [53]. All videos were coded blindly by the same person. The whole trial duration (one week) was coded, except when the clutch totally disappeared because all tadpoles were transported, eaten, or dead.

For each male, we coded whether he cannibalised (yes/no) and/or transported tadpoles (yes/no) of the focal clutch. A male was considered to perform transport if he picked up at least one tadpole on his back and left the clutch location with it (see Movie S1). A male was considered to have cannibalised tadpoles, if he ate at least one larva from the clutch (see Movie S2). If the male did neither of these two behaviours during the entire trial, it was coded as ignored. In most of the cases, transport or cannibalism was not performed on the entire clutch, because occasionally single eggs failed to develop or died from other causes before the males performed the recorded behaviour. Occasionally, males also lost a few tadpoles during their transport. In these cases, the final number of tadpoles deposited or consumed was lower than the total number of tadpoles at the start of the trial. We therefore coded the presence/absence of the behaviours as such. We also recorded the day on which each behaviour first occurred.

### Statistical analysis

Statistical analysis was performed in R [54] using the RStudio software [55]. In order to reveal if there were significant differences in the likelihood to perform tadpole transport across conditions, we fitted three generalised linear models (GLMs), with respectively transport, cannibalism and ignore as binomial response variables. All three models use the same fixed effects, the experimental condition and the age of the male. As each frog was tested only once, we did not include any random effects. Then, we ran an analysis of variance (function Anova from package car [56]) on our models to see if there was a significant difference in transport, cannibalism or ignore between conditions, and if any other explanatory variable had a significant effect on transport, cannibalism or ignore likelihood. Afterwards, if the experimental condition had a significant effect, we performed Tukey post-hoc pairwise comparisons (with pairs(emmeans()), from package emmeans [57]) to see if there were statistically significant differences between conditions. Additionally, to determine whether males first transported or cannibalised tadpoles in the trials, we performed a Wilcoxon rank sum test.

## Results

**Fig. 2:**
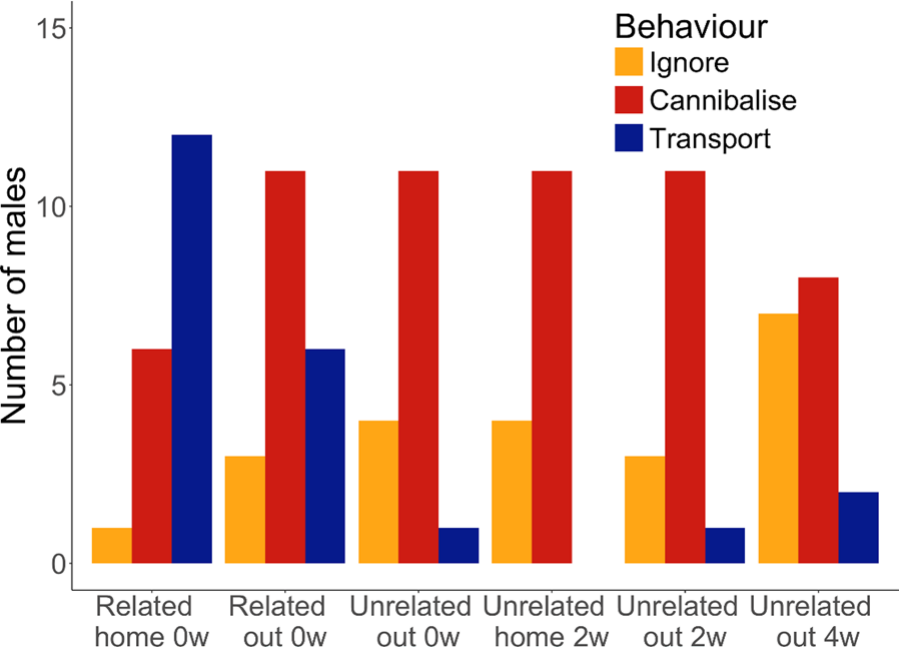
Number of males observed ignoring, cannibalising, and transporting tadpoles from the focal clutches in each condition. N = 15 males in each condition

The present study investigates how territory novelty and mating activity shape tadpole transport and cannibalism in *A. femoralis* males. In a laboratory experiment, the occurrence of tadpole transport and cannibalistic behaviour were recorded, then compared across six conditions. The number of males that performed tadpole transport varied between 0 and 12 out of 15 in a given condition. The number of males that cannibalised tadpoles varied between 6 and 11, and the number of males ignoring the clutch varied between 1 and 7 (Fig. 2). Except for the condition Unrelated home 2w, where no males transported, the three behaviours are present in each condition.

**Fig. 3:**
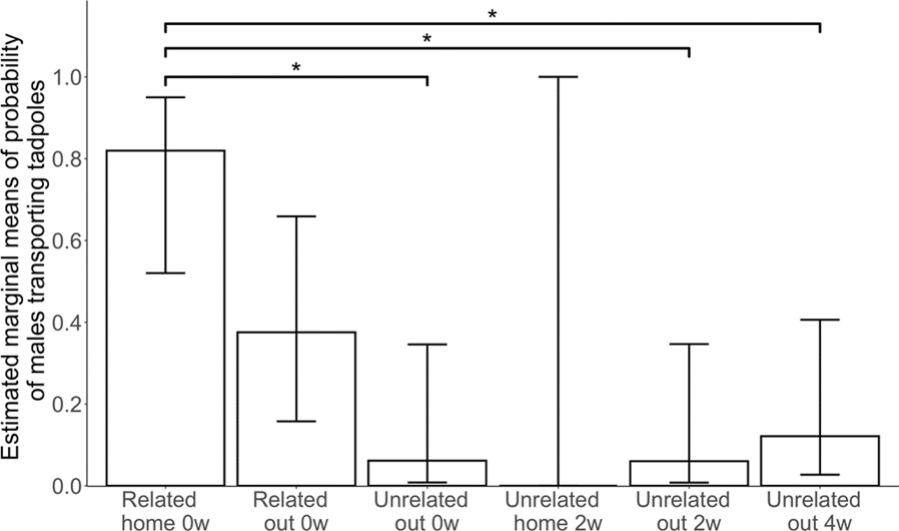
Estimated marginal means of probability of males transporting tadpoles in each condition. Statistically significant post-hoc pairwise comparisons (< 0.05) are indicated using an asterisk. The top of the bar is the mean estimated by the model. Error bars represent confidence intervals for the estimated marginal means

Only “experimental condition” had a statistically significant effect on the likelihood of tadpole transport to occur (Analysis of Deviance Table, Chisq = 33.56, df = 5, *p* < 0.001), while the age of the male and the month the experiment was conducted did not (Table 1). The post-hoc pairwise comparisons (Table 2) showed that males in their own territory with their own clutch were significantly more likely to perform tadpole transport than males in a new territory with an unrelated clutch (Related home 0w vs. Unrelated out 0w, Tukey, z = 3.12, *p* = 0.022, Fig. 3). Males in their own territory with their own clutch also transported significantly more than males in a new territory which were given an unrelated clutch after two weeks of isolation (Related home 0w vs. Unrelated out 2w, Tukey, z = 3.088, *p* = 0.028, Fig. 3), as well as after four weeks of isolation (Related home 0w vs. Unrelated out 4w, Tukey, z = 2.95, *p* = 0.038, Fig. 3).

**Table 1:** Table of results of analysis of variance tests for the three GLM models, with the Chi-squared statistic (Chisq), degrees of freedom (df) and p-value (p). Significant p-values (< 0.05) are indicated with an asterisk

Response variable	Explanatory variable	Chisq	Df	p
Transport	Condition	33.56	5	< 0.001 *
	Age	0.16	1	0.69
Cannibalism	Condition	4.96	5	0.42
	Age	0.20	1	0.66
Ignore	Condition	6.54	5	0.26
	Age	0.007	1	0.94

**Table 2:** Table of the post-hoc pairwise comparisons of the model with “transport” as response variable. Comparison indicates the two experimental conditions that are being compared; z is the z.ratio; df is the degree of freedom; p is the p value. Significant *p* values (< 0.05) are indicated with an asterisk

Comparison	z	p
Related home 0w—Related out 0w	1.95	0.37
Related home 0w—Unrelated out 0w	3.12	0.022 *
Related home 0w—Unrelated home 2w	0.01	1
Related home 0w—Unrelated out 2w	3.08	0.025 *
Related home 0w—Unrelated out 4w	2.95	0.038 *
Related out 0w—Unrelated out 0w	1.91	0.40
Related out 0w—Unrelated home 2w	0.01	1
Related out 0w—Unrelated out 2w	1.93	0.39
Related out 0w—Unrelated out 4w	1.59	0.61
Unrelated out 0w—Unrelated home 2w	0.01	1
Unrelated out 0w—Unrelated out 2w	0.01	1
Unrelated out 0w—Unrelated out 4w	− 0.58	0.99
Unrelated home 2w—Unrelated out 2w	− 0.01	1
Unrelated home 2w—Unrelated out 4w	− 0.01	1
Unrelated out 2w—Unrelated out 4w	− 0.60	0.99

Neither the experimental condition, nor the age of the males and the month the experiment was conducted had a statistically significant effect on cannibalism rates (Table 1). Cannibalistic behaviour was indeed observed across all experimental conditions (Fig. 2), although the lowest occurrences were observed in the “Related home 0w” condition.

Neither the experimental condition, nor the age of the males and the month the experiment was conducted had a statistically significant effect on the level of ignoring the clutch (Table 1).

**Fig. 4:**
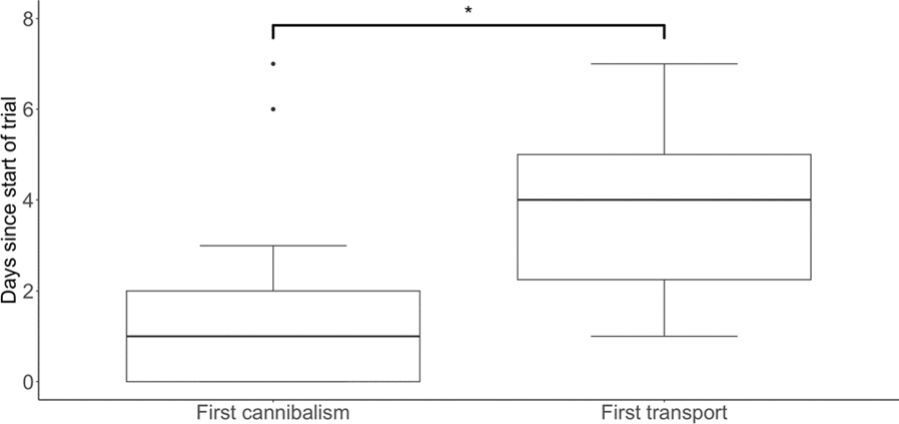
Time since the start of trial to first observation of cannibalism or transport, in all conditions. Statistically significant difference (< 0.05) is indicated using an asterisk

Looking at the time in days of the first observation of cannibalism and of transport, there was a statistically significant difference between these two behaviours (Wilcoxon rank sum test, W = 138.5, *p* < 0.001), regardless of the condition. While cannibalism mainly occurred early after starting a trial (mean = 1.22, sd = 1.51), transport occurred on average later in the trial (mean = 3.82, sd = 1.62). We also observed a wide range of variation in the time it took for males to initiate tadpole transport, from one to seven days after the start of the trial (Fig. 4). In contrast, the majority of males exhibited cannibalistic behaviour within the first two days following the start of the trial, while four individuals cannibalised on the third day and three on the sixth and seventh days after the start of the trial.

It is interesting to note that twelve males both cannibalised and later transported the remaining tadpoles. Four of these males were in the condition “Related home 0w”, five in “Related out 0w”, one in “Unrelated out 0w” and two in “Unrelated out 4w”.

## Discussion

Our study reveals novel insight into the factors shaping tadpole transport and cannibalism in male poison frogs during territory take-over. Our results partially confirm the results from previous studies [40, 50] and indicate that multiple factors are independently influencing these behaviours in male *A. femoralis*.

The decision rule “transport all clutches inside and eat all clutches outside my territory” [40] for *A. femoralis* might actually be more complex than previously thought. Our study shows that the likelihood to transport tadpoles was shaped by territory ownership, relatedness to clutches, and possibly also by recent mating activity. However, our results do not clearly disentangle which of these factors influenced cannibalistic behaviours. Therefore, tadpole transport and cannibalism seem to be influenced independently by the tested factors, which means that factors facilitating tadpole transport do not necessarily inhibit cannibalistic behaviours and the other way around.

Our study shows that males in their own territory with their own clutch were more likely to transport tadpoles than males in a new territory with an unrelated clutch. Interestingly, no males transported tadpoles when they were given an unrelated clutch in their own territory after two weeks of isolation. This result contrasts findings from previous studies in which males transported most unrelated clutches in their own territory [40, 50]. The difference in the results between the present and the previous study could be due to differences in the timing since last mating. Both in the previous study and in this one, only the timeframe during which mating was prevented (i.e. isolation of males), but not the actual duration since last mating, was precisely assessed and controlled for. Therefore, we cannot exclude the possibility that “time since last mating event” was the underlying factor that resulted in the contrasting results of both studies. In future experiments, the actual time since last mating should be assessed and incorporated in the study design, to clearly identify the importance of the time since last mating activity on reproductive behaviours.

In a variety of animal species, including ants, cichlid fish, rodents, goats, sheep, bats and primates, parents rely on offspring-specific cues to discriminate between their own and conspecific unrelated offspring (reviewed in [58]). The results of this and previous studies [40, 50, 59] however demonstrate that male poison frogs do not discriminate between their own and unrelated clutches exclusively based on clutch-specific cues, such as odour or visual information; even though olfaction plays a major role in *A. femoralis* for selecting suitable pools for tadpole deposition [60, 61].

In our study, we did not find significant differences in the prevalence of cannibalism across conditions, however the lowest levels of cannibalism were observed in the condition where the male was not given an isolation period of two weeks and tested in its own territory. This suggests that recent mating activity and/or close spatial proximity to a female reduces the rate of cannibalism in males. Studies in rats have shown that males are less infanticidal immediately after offspring birth, and that the level of infanticide increases with the increase of time since mating [62]. Similar results have been found in house wrens, where males are less likely to destroy eggs in their own nest right after mating [15]. Similar patterns might also occur in frogs, and future studies should look into the endocrine regulation of infanticidal versus parental behaviours.

Time spent in a new territory did not have a significant impact on the level of cannibalism. Contrary to our expectation, levels of cannibalism did not decrease with time spent in a new territory. However, by increasing the time spent in a new territory, we also increased the time since the last mating, as we did not allow them to mate during this time. We were therefore not able to clearly disentangle these factors. Surprisingly, some males even cannibalized own clutches inside their own territory. Maybe this was a result from the handling of the own clutch, although similar handling did not show such an effect in a previous study [40]. Alternative explanations for this behaviour could be differences in the levels of aggression among males [63] or increased energetic demand of some individuals.

Interestingly, some males both cannibalised and transported tadpoles of the same clutch. Even though this phenomenon was already observed in a previous study [40], the underlying reasons and mechanisms remain unclear. In one of these cases, we observed that one tadpole climbed on the male’s back while the male was cannibalising the rest of the clutch. We assume that the presence of a tadpole on the male’s back triggered tadpole transport [64]. Our study also showed that males cannibalise clutches during the first few days of the trial, whereas transport usually takes place a few days later. It therefore seems that the decision-making process of whether to cannibalise or not is very quick. The variation in timing of tadpole transport was much larger, raising questions about relevant internal (e.g. hormonal state, internal clock) and external (e.g. visual cues of developmental stage of clutches) cues that trigger tadpole transport behaviour in poison frogs (cf. 59). Alternatively, these observations could indicate that transport and cannibalism are two independent processes that are triggered by different factors. Taking over a new territory could trigger cannibalistic behaviours, while time since last mating could influence the likelihood of tadpole transport.

The present study revealed considerable variation in the males’ behaviours, even within the same treatment. This variation may be due to the complex decision-making process involved in balancing parental care and aggression linked to territorial defence. Individual males may place varying degrees of importance on each of these cues, thus resulting in different behavioural phenotypes. Indeed, previous studies in *A. femoralis* have found consistent individual differences in individual levels of boldness, exploration, and territorial aggression [65, 66]. Personality differences exist already at the tadpole stage and are maintained over metamorphosis [67], and are linked to reproductive success, such as the number of mating partners, number of mating events, or offspring survival [63]. The impact of personality on mating and parental behaviour is found across a wide range of animal taxa. In the common goby, personality predicts filial cannibalism [20] and nest allocation in males [68]. In the Eurasian perch, individuals that were more asocial and inactive were more cannibalistic than the more social and active individuals [69]. Further studies should investigate the role of personality on parental and infanticidal behaviour in *A. femoralis*.

Contrary to what we expected, we found that males ignored the clutch to a similar extent across all conditions. Perhaps some males simply did not manage to transport the clutch in the given timeframe of the experiment, but would have done so if given more time. Indeed, there is some variability in the time at which males start to transport, with several males transporting at the very end of the experiment. However, in most of these trials, males did not even inspect the clutches. It is possible that some males were unaware of the presence of the clutch in their territory, although clutches were always placed in a conspicuous location at the front of the terrarium. Alternatively, males may simply ignore clutches with unclear paternity status, to avoid spending energy to care for it. Indeed, offspring abandonment is frequently associated with filial cannibalism and may represent an evolutionary advantage if its benefits exceed those of caring for the offspring [70].

## Conclusions

In summary, our study shows that tadpole transport and clutch cannibalism are shaped by different factors in males of the poison frog *Allobates femoralis*. Our results confirm previous findings that males differentiate their offspring using territorial recognition as a proxy. Recent mating promotes transport in the male’s territory, while factors that promote or inhibit cannibalistic behaviour remain unclear. Our findings suggest that transport and cannibalism are two independent processes, and factors influencing one behaviour do not necessarily affect the other. The decision-making processes involved in parental care and cannibalism therefore appear to be more complex than what was previously thought. Further studies should investigate the hormonal and neural mechanisms shaping parental care and infanticidal behaviours. In summary, our study demonstrated that territory ownership and relatedness to the clutch, but possibly also reproductive state influenced parental behaviours in poison frogs.

## Supplementary Information

Additional file 1.

Additional file 2.

## Data Availability

The datasets generated and analysed during the current study as well as the code used for analysis are available in the Open Science framework repository, 10.17605/OSF.IO/PCZKF.
